# Characteristics of Coronavirus Disease in Allogeneic Hematopoietic Stem Cell Transplantation During the Omicron Wave: A Single-Center Study

**DOI:** 10.1093/ofid/ofae038

**Published:** 2024-01-23

**Authors:** Panpan Zhu, Luxin Yang, Lizhen Liu, Xiaoyu Lai, Jimin Shi, Yanmin Zhao, Jian Yu, Huarui Fu, Yishan Ye, Yibo Wu, He Huang, Yi Luo

**Affiliations:** Bone Marrow Transplantation Center of The First Affiliated Hospital & Liangzhu Laboratory, Zhejiang University School of Medicine, Hangzhou, China; Institute of Hematology, Zhejiang University, Hangzhou, China; Zhejiang Province Engineering Laboratory for Stem Cell and Immunity Therapy, Hangzhou, China; Bone Marrow Transplantation Center of The First Affiliated Hospital & Liangzhu Laboratory, Zhejiang University School of Medicine, Hangzhou, China; Institute of Hematology, Zhejiang University, Hangzhou, China; Zhejiang Province Engineering Laboratory for Stem Cell and Immunity Therapy, Hangzhou, China; Bone Marrow Transplantation Center of The First Affiliated Hospital & Liangzhu Laboratory, Zhejiang University School of Medicine, Hangzhou, China; Institute of Hematology, Zhejiang University, Hangzhou, China; Zhejiang Province Engineering Laboratory for Stem Cell and Immunity Therapy, Hangzhou, China; Bone Marrow Transplantation Center of The First Affiliated Hospital & Liangzhu Laboratory, Zhejiang University School of Medicine, Hangzhou, China; Institute of Hematology, Zhejiang University, Hangzhou, China; Zhejiang Province Engineering Laboratory for Stem Cell and Immunity Therapy, Hangzhou, China; Bone Marrow Transplantation Center of The First Affiliated Hospital & Liangzhu Laboratory, Zhejiang University School of Medicine, Hangzhou, China; Institute of Hematology, Zhejiang University, Hangzhou, China; Zhejiang Province Engineering Laboratory for Stem Cell and Immunity Therapy, Hangzhou, China; Bone Marrow Transplantation Center of The First Affiliated Hospital & Liangzhu Laboratory, Zhejiang University School of Medicine, Hangzhou, China; Institute of Hematology, Zhejiang University, Hangzhou, China; Zhejiang Province Engineering Laboratory for Stem Cell and Immunity Therapy, Hangzhou, China; Bone Marrow Transplantation Center of The First Affiliated Hospital & Liangzhu Laboratory, Zhejiang University School of Medicine, Hangzhou, China; Institute of Hematology, Zhejiang University, Hangzhou, China; Zhejiang Province Engineering Laboratory for Stem Cell and Immunity Therapy, Hangzhou, China; Bone Marrow Transplantation Center of The First Affiliated Hospital & Liangzhu Laboratory, Zhejiang University School of Medicine, Hangzhou, China; Institute of Hematology, Zhejiang University, Hangzhou, China; Zhejiang Province Engineering Laboratory for Stem Cell and Immunity Therapy, Hangzhou, China; Bone Marrow Transplantation Center of The First Affiliated Hospital & Liangzhu Laboratory, Zhejiang University School of Medicine, Hangzhou, China; Institute of Hematology, Zhejiang University, Hangzhou, China; Zhejiang Province Engineering Laboratory for Stem Cell and Immunity Therapy, Hangzhou, China; Bone Marrow Transplantation Center of The First Affiliated Hospital & Liangzhu Laboratory, Zhejiang University School of Medicine, Hangzhou, China; Institute of Hematology, Zhejiang University, Hangzhou, China; Zhejiang Province Engineering Laboratory for Stem Cell and Immunity Therapy, Hangzhou, China; Bone Marrow Transplantation Center of The First Affiliated Hospital & Liangzhu Laboratory, Zhejiang University School of Medicine, Hangzhou, China; Institute of Hematology, Zhejiang University, Hangzhou, China; Zhejiang Province Engineering Laboratory for Stem Cell and Immunity Therapy, Hangzhou, China; Bone Marrow Transplantation Center of The First Affiliated Hospital & Liangzhu Laboratory, Zhejiang University School of Medicine, Hangzhou, China; Institute of Hematology, Zhejiang University, Hangzhou, China; Zhejiang Province Engineering Laboratory for Stem Cell and Immunity Therapy, Hangzhou, China

**Keywords:** allogeneic hematopoietic stem cell transplantation, anti-thymocyte globulin, COVID-19, early post-transplantation, omicron wave

## Abstract

**Objectives:**

This study aimed to characterize the clinical characteristics, outcomes, and risk factors for coronavirus disease 2019 (COVID-19) in 492 patients who underwent allogeneic hematopoietic stem cell transplantation (allo-HSCT) during the Omicron wave.

**Methods:**

Data were retrospectively collected from patient charts and the electronic medical record systems at the First Affiliated Hospital of Zhejiang University School of Medicine between December 2022 and January 2023.

**Results:**

The median follow-up period of the entire cohort was 62 days. Myeloid malignancies (58.5%) and acute lymphocytic leukemia (30.5%) constituted the most common underlying disease. Among the 492 patients, 415, 67, and 10 exhibited mild, moderate, and severe COVID-19, respectively. The incidence of moderate-to-severe COVID-19 was 15.7%. The 60-day overall survival and complete resolution rates were 98.1% and 80.6%, respectively. The risk factors for moderate-to-severe COVID-19 included corticosteroid use within 3 months before diagnosis, <6 months interval between allo-HSCT and COVID-19 diagnosis, and antithymocyte globulin use for graft-versus-host disease prophylaxis.

**Conclusions:**

During the Omicron wave, patients with allo-HSCT demonstrated a low COVID-19–related mortality rate and high moderate-to-severe and prolonged disease incidence. Prevention in the early posttransplantation period is critical for allo-HSCT recipients receiving corticosteroids.

## Teaser

We present the clinical characteristics, outcomes, and risk factors for coronavirus disease 2019 (COVID-19) in allogeneic hematopoietic stem cell transplantation recipients during the Omicron wave. The recipients exhibited a low COVID-19–related mortality rate and high moderate-to-severe and prolonged disease incidence.

Coronavirus disease 2019 (COVID-19) is caused by the severe acute respiratory syndrome coronavirus 2 (SARS-CoV-2). The World Health Organization declared COVID-19 a global pandemic on 11 March 2020 [1, 2]. On 26 November 2021, the World Health Organization designated Omicron, B.1.1.529, as a new variant of concern, which would change the trajectory of the COVID-19 pandemic [3]. Being the most heavily mutated variant, the Omicron variant exhibits enhanced transmissibility and resistance to COVID-19 vaccine-induced immunity [4–6]. The Omicron/BA.5 subvariant has become dominant globally, including in China [7, 8]. As of 16 March 2023, >760 million cases of COVID-19 have been confirmed worldwide, including >6 million deaths [9]. The significant risk factors for COVID-19–related morbidity and mortality included older age, diabetes, hypertension, cancer, and hematological malignancies [10, 11].

Allogeneic hematopoietic stem cell transplantation (allo-HSCT) is a potentially curative option for patients with various hematological disorders [12, 13]. The recipients of allo-HSCT are often immunocompromised and have a high risk of infection [14, 15]. In particular, patients with hematological malignancies have a higher risk of developing severe COVID-19 compared with those without hematological malignancies [16–18]. During the pre-Omicron wave, these patients reportedly exhibited a high risk of COVID-19–related severity and mortality, with a 30-day overall survival (OS) rate of 68% to 73% [19, 20]. Moreover, COVID-19 cases with the Omicron variant in the general and immunocompromised populations demonstrated a low COVID-19–related morbidity and mortality [21–24]. However, information regarding the impact of COVID-19 infection on allo-HSCT recipients during the Omicron wave is limited. Therefore, this study aimed to characterized the clinical characteristics, outcomes, and risk factors for COVID-19 in recipients of allo-HSCT during the Omicron wave.

## METHODS

### Study Design

Patients aged ≥18 years and diagnosed with COVID-19 between December 2022 and January 2023 at the First Affiliated Hospital of Zhejiang University School of Medicine were included. Data were retrospectively collected from patient charts and electronic medical record systems. Patients who received allo-HSCT were diagnosed via antigen or SARS-CoV-2 reverse transcription polymerase chain reaction test using nasopharyngeal swab samples. There are 3 types of COVID-19 vaccines: an inactivated COVID-19 vaccine (vero cell), a recombinant subunit COVID-19 vaccine (CHO cell), and an adenovirus vector COVID-19 vaccine (Ad5).

### Patient Consent Statement

All patients provided written informed consent for data collection. This study was performed in accordance with the Declaration of Helsinki and approved by the Ethics Review Committee of the First Affiliated Hospital of Zhejiang University School of Medicine (Approval No. IIT20230307A).

### Transplantation Procedures

Patients underwent myeloablative or reduced intensity conditioning. All conditioning regimens have been described in a previous study [25]. Peripheral blood stem cells without ex vivo T-cell depletion were collected from donors treated with rhG-CSF (filgrastim; Kirin, Japan; 5–7.5 μg/kg per day). Prophylaxis for graft-versus-host disease (GVHD) comprised cyclosporine A, methotrexate, and low-dose mycophenolate mofetil. Rabbit antithymocyte globulin (ATG-G; thymoglobulin, Genzyme, Cambridge, MA; 6 mg/kg total dose) or ATG-F (Fresenius, Bad Homburg, Germany; 10 mg/kg total dose) was administered to allo-HSCT recipients using haploidentical donors. However, for allo-HSCT recipients with unrelated donors, the total dose of ATG-G was 6 mg/kg. A small portion of allo-HSCT recipients with human leukocyte antigen–matched sibling donors was treated with ATG-G (total dose, 4.5 mg/kg). Calcineurin inhibitors, corticosteroids, tyrosine kinase inhibitors (eg, ruxolitinib), and methotrexate were used for patients with GVHD.

### Definitions

The incidence of moderate-to-severe COVID-19 at the end of the follow-up period was the primary outcome. COVID-19 severity was classified as mild (no supplemental oxygen needed), moderate (supplemental oxygen needed), or severe (mechanical ventilation required) [20]. The proportion of patients with moderate-to-severe COVID-19 compared with the total number of patients with COVID-19 during the observation period was used to define the incidence of moderate-to-severe COVID-19. The time from the day of onset of COVID-19–related clinical manifestations or a positive polymerase chain reaction or antigen test to the day of infection resolution or the last follow-up was used to define the duration of the COVID-19. No clinical signs/symptoms of COVID-19 regardless of microbiological tests via nasopharyngeal swab samples were used to define infection resolution. SARS-CoV-2 infection status was described as ongoing (no clinical signs of improvement), improved (continued treatment despite resolution of COVID-19 signs/symptoms), or resolved (no COVID-19 signs/symptoms and completion of planned treatment) [20]. Asymptomatic patients with COVID-19 were defined as those whose clinical COVID-19–related signs/symptoms resolved without microbiological treatment.

### Statistical Analysis

The demographic and clinical characteristics of patients with COVID-19 who had received allo-HSCT were analyzed. Continuous variables are presented as median (range). Nonnormally distributed continuous variables were compared using the Mann–Whitney *U* test. Categorical variables are expressed as frequencies (percentages) and were compared using Pearson χ^2^ test (T ≥ 5 and n ≥ 40), χ^2^ test with continuity correction (1 ≤ T < 5 and n ≥ 40), and Fisher exact test. Survival analysis was performed using the Kaplan–Meier method. The risk factors for moderate-to-severe COVID-19 were assessed using univariate and multivariate logistic regression analyses. Variables with a *P* value of ≤.1 were considered for multivariable analysis. The hazard ratios of COVID-19 survival were estimated using Cox regression models. All analyses were performed using SPSS version 26.0 (SPSS, IBM Corp., Chicago, IL, USA) and R language statistical software (http://www.r-project.org). A 2-tailed significance *P* level <.05 was used.

## RESULTS

### Characteristics of Transplantation

A total of 732 adult patients who received allo-HSCT were followed up between December 2022 and January 2023. Among them, 492 patients (245 men and 247 women) were diagnosed with COVID-19 after HSCT. Supplementary Table 1 shows the baseline clinical characteristics of patients with COVID-19. The median follow-up period was 62 (6–110) days after COVID-19 diagnosis. The median age at COVID-19 diagnosis was 41 (18–71) years. The median body mass index was 21.7 (13.0–41.4) kg/m^2^. Myeloid malignancy (n = 288; 58.5%) and acute lymphocyte leukemia (n = 150; 30.5%) were the most common underlying diseases. Most patients (75.2%) underwent allo-HSCT after 2020. Donor types included 88 (17.0%) matched sibling donors, 344 (70.0%) haploidentical donors, and 64 (13.0%) unrelated donors. Myeloablative conditioning regimen was administered to 86.6% allo-HSCT recipients. ATG was administered to 430 (87.4%) patients for GVHD prophylaxis.

### COVID-19 Characteristics

The median interval between allo-HSCT and COVID-19 diagnosis of the overall population was 18.5 (0.8–145.8) months, with 17.5% of patients with confirmed COVID-19 diagnosis within 6 months after HSCT. Approximately 25.4% patients received COVID-19 vaccines before COVID-19 diagnosis, with a median period from vaccination to COVID-19 diagnosis of 456 (2–694) days. Among 125 patients who received COVID-19 vaccines, 94 received vaccines before allo-HSCT, with a median interval from vaccination to allo-HSCT of 267.5 (22–560) days. Overall, 31 were vaccinated after allo-HSCT, with a median interval from allo-HSCT to vaccination of 1666 (range, 117–3828) days. Furthermore, 18 received 1 dose of COVID-19 vaccine, 67 received 2 doses of COVID-19 vaccine, and 40 received a third dose as a booster (Supplementary Table 1).

Approximately 28.3% allo-HSCT recipients experienced GVHD at the time of COVID-19 diagnosis. Approximately 34.6% patients who had COVID-19 within 3 months before diagnosis were administered with corticosteroids, and 50.8% patients were on immunosuppressive treatment 3 months before COVID-19 diagnosis. A total of 415, 67, and 10 patients exhibited mild, moderate, and severe COVID-19, respectively. The incidence of moderate-to-severe COVID-19 in allo-HSCT recipients was 15.7%. The characteristics of patients with mild and moderate-to-severe COVID-19 are presented in Table 1.

**Table 1.** Baseline Clinical Characteristics of Allo-HSCT Recipients With COVID-19

	Mild (N = 415)	Moderate-to-Severe (N = 77)	*P* Value
Age at COVID-19 diagnosis, median (range), y	41 (18–71)	47 (20–71)	.015
18–29	91 (21.9)	10 (13.0)	.271
30–39	103 (24.8)	17 (22.1)	
40–49	103 (24.8)	20 (26.0)	
50–59	86 (20.7)	22 (28.6)	
≥60	32 (7.7)	8 (10.4)	
Sex, male/female	203/212	42/35	.364
BMI, median (range), kg/m^2^	21.8 (13.0–40.0)	21.0 (13.3–41.4)	.059
<18.5	64 (15.4)	20 (26.0)	.050
18.5–23.9	242 (58.3)	43 (55.8)	
≥24.0	109 (26.3)	14 (18.2)	
Underlying disease			.364
AML/MDS	241 (58.1)	47 (61.0)	
ALL	131 (31.6)	19 (24.7)	
Other	43 (10.4)	11 (14.3)	
Donor type			.070
MSD	76 (18.3)	8 (10.4)	
HID	290 (69.9)	54 (70.1)	
URD	49 (11.8)	15 (19.5)	
Conditioning regimens			.005
MAC	367 (88.4)	59 (76.6)	
RIC	48 (11.6)	18 (23.4)	
ATG for GVHD prophylaxis			.012
Yes	356 (85.8)	74 (96.1)	
No	59 (14.2)	3 (3.9)	
ATG type (dosage)			.045
ATG-G (6 mg/kg)	271	59	
ATG-G (4.5 mg/kg)	17	5	
ATG-F (10 mg/kg)	68	10	
Year of transplantation			.001
Before 2019	114 (27.5)	8 (10.4)	
After 2020	301 (72.5)	69 (89.6)	
Interval from allo-HSCT to COVID-19 diagnosis, median (range), mo	19.3 (0.8–145.8)	9.8 (1.0–58.2)	<.001
<6	60 (14.5)	26 (33.8)	<.001
6–12	67 (16.1)	15 (19.5)	
12–24	119 (28.7)	19 (24.7)	
≥24 m	169 (40.7)	17 (22.1)	
On immunosuppression treatment within 3 mo before COVID-19 diagnosis			.003
Yes	199 (48.0)	51 (66.2)	
No	216 (52.0)	26 (33.8)	
On corticosteroid therapy within 3 mo before COVID-19 diagnosis			<.001
Yes	129 (31.1)	41 (53.2)	
No	286 (68.9)	36 (46.8)	
GVHD at COVID-19 diagnosis			.371
Yes	114 (27.5)	25 (32.5)	
No	301 (72.5)	52 (67.5)	
History of acute GVHD			.037
Yes	113 (27.2)	30 (39.0)	
No	302 (72.8)	47 (61.0)	
History of grade II–IV acute GVHD			.103
Yes	61 (14.7)	17 (22.1)	
No	354 (85.3)	60 (77.9)	
History of grade III–IV acute GVHD			.264
Yes	17 (4.1)	6 (7.8)	
No	398 (95.9)	71 (92.2)	
History of chronic GVHD			.056
Yes	200 (48.2)	28 (36.4)	
No	215 (51.8)	49 (63.6)	
History of moderate-to-severe chronic GVHD			.769
Yes	75 (18.1)	15 (19.5)	
No	340 (81.9)	62 (80.5)	
History of COVID-19 vaccination			.487
Yes	103 (24.8)	22 (28.6)	
No	312 (75.2%	55 (71.4)	
Time from the last vaccination to COVID-19 diagnosis, median (range), days	456 (2–694)	455.5 (13–618)	.775
COVID-19–directed therapies			
Azvudine	2	4	<.001
Molnupiravir	4	7	
Paxlovid	5	23	
Other antivirals	0	1	
None	404	42	

Abbreviations: ALL, acute lymphoblastic leukemia; allo-HSCT, allogeneic hematopoietic stem cell transplantation; AML, acute myelocytic leukemia; ATG, anti-thymocyte globulin; BMI, body mass index; COVID-19, coronavirus disease 2019; GVHD, graft-versus-host disease; HID, haploidentical donor; MAC, myeloablative conditioning; MDS, myelodysplastic syndrome; MSD, matched sibling donor; RIC, reduced intensity conditioning; URD, unrelated donor.

Patients with moderate-to-severe COVID-19 were older (47 vs 41 years, *P* = .015) and received ATG for GVHD prophylaxis more frequently than those with mild COVID-19 (96.1% vs 85.5%, *P* = .012). Patients with mild COVID-19 had a longer interval from allo-HSCT to COVID-19 diagnosis than those with moderate-to-severe COVID-19 (19.3 vs 9.8 months, *P* < .001). Compared with patients with mild COVID-19, more patients with moderate-to-severe COVID-19 received immunosuppressive treatment (*P* = .003) or corticosteroids (*P* < .003) 3 months before COVID-19 diagnosis. In addition, 39.0% patients with moderate-to-severe COVID-19 and 27.2% of those with mild COVID-19 had a history of acute GVHD (*P* = .037). In the univariate analysis (Table 2), immunosuppressive or corticosteroid use within 3 months before COVID-19 diagnosis, interval from allo-HSCT to COVID-19 diagnosis, ATG for GVHD prophylaxis, conditioning regimens, donor types, and history of acute GVHD were significant factors for moderate-to-severe COVID-19. In the multivariate analysis (Table 2), corticosteroid use within 3 months before COVID-19 diagnosis (odds ratio [OR], 2.115; 95% confidence interval [CI], 1.262–3.546; *P* = .004), <6-month interval between allo-HSCT and COVID-19 diagnosis (OR, 2.23; 95% CI, 1.259–3.969; *P* = .006), and ATG for GVHD prophylaxis (OR, 3.450; 95% CI, 1.040–11.444; *P* = .043) were significant risk factors for moderate-to-severe COVID-19.

**Table 2.** Univariate and Multivariate Analyses for Moderate-to-Severe COVID-19

	Univariate Analysis			Multivariate Analysis		
	OR	95% CI	*P* Value	OR	95% CI	*P* Value
On corticosteroid therapy 3 mo before COVID-19 diagnosis (Ref. without)	2.525	1.541–4.136	<.001	2.115	1.262–3.456	.004
Interval from allo-HSCT to COVID-19 diagnosis (Ref. ≥6 mo)	3.016	1.748–5.206	<.001	2.234	1.259–3.963	.006
ATG for GVHD prophylaxis (Ref. without)	4.088	1.248–13.394	.020	3.450	1.040–11.444	.043
Age at COVID-19 diagnosis (Ref. <50 y)	1.697	.969–2.663	.066	…	…	.351
BMI						
<18.5	1 (ref)	1 (ref)		1 (ref)	1 (ref)	…
18.5–23.9	0.569	.313–1.034	.064	…	…	.602
≥24.0	0.411	.194–.870	.020	…	…	.223
Conditioning regimens (Ref. MAC)	0.429	.234–.787	.006	…	…	.098
Donor type						
URD	1 (ref)	1 (ref)		1 (ref)	1 (ref)	…
HID	0.608	.318–1.162	.132	…	…	.118
MSD	0.344	.136–.872	.024	…	…	.373
History of acute GVHD (Ref. without)	1.796	1.028–2.831	.039	…	…	.173
History of chronic GVHD (Ref. without)	0.612	.372–1.015	.057	…	…	.247
On immunosuppression treatment within 3 m before COVID-19 diagnosis (Ref. without)	2.129	1.278–3.546	.004	…	…	.667

Abbreviations: allo-HSCT, allogeneic hematopoietic stem cell transplantation; ATG, antithymocyte globulin; CI, confidence interval; COVID-19, coronavirus disease 2019; GVHD, graft-versus-host disease; HID, haploidentical donor; MAC, myeloablative conditioning; MSD, matched sibling donor; OR, odds ratio; URD, unrelated donor.

### Immune Cells and Response to COVID-19

**Figure 1.**
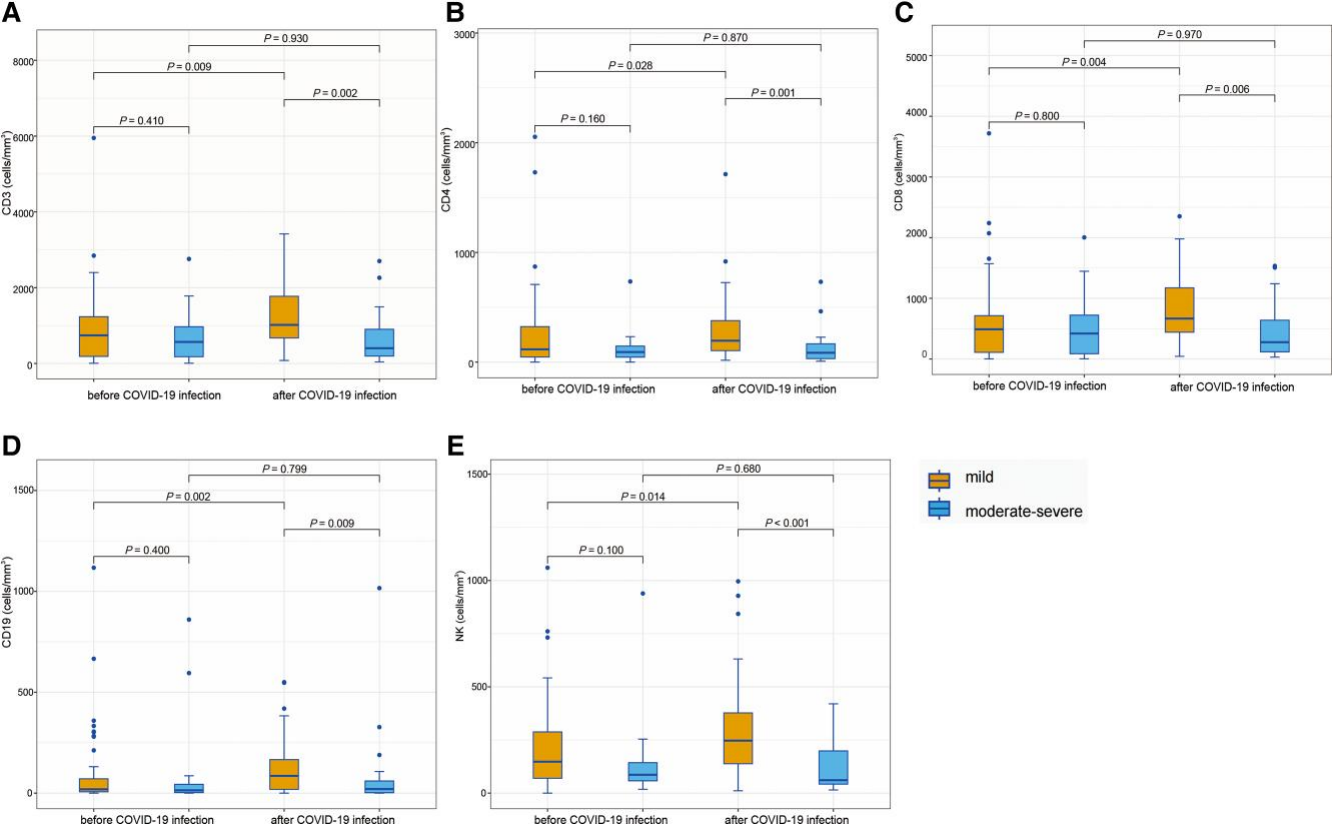
Immune cell levels in patients with allo-HSCT before and after COVID-19 diagnosis. The 2 bars on the left show the immune cell levels before COVID-19 diagnosis. The 2 bars on the right represent the immune cell levels following COVID-19 diagnosis. CD3^+^ T cells (*A*), CD4^+^ T cells (*B*), CD8^+^ T cells (*C*), CD19^+^ cells (*D*), and natural killer cells (*E*). allo-HSCT, allogeneic hematopoietic stem cell transplantation; COVID-19, coronavirus disease 2019.

Peripheral immune cells (CD3^+^ T, CD4^+^ T, CD8^+^ T, natural killer, and CD19^+^ T) were detected following allo-HSCT in 76 patients with COVID-19 (56 with mild disease and 20 with moderate-to-severe disease; Supplementary Table 2). Immune cells were detected at a median of 28.5 (2–171) and 39 (2–79) days before and after COVID-19 diagnosis, respectively. The levels of various immune cells before COVID-19 diagnosis were similar between the mild and moderate-to-severe groups. Following COVID-19 diagnosis, patients with moderate-to-severe disease exhibited significantly lower immune cell count than those with mild disease (Figure 1). In the mild group, the preinfection levels of various immune cells were significantly lower than the postinfection levels. However, no significant difference was observed in the immune cell status in patients with moderate-to-severe COVID-19.

Conversely, 81 allo-HSCT recipients were tested for antibodies to SARS-CoV-2 at a median interval of 44 (6–80) days after COVID-19 diagnosis. Among the 81 patients, 68 were positive and 3 were weakly positive for immunoglobulin G. Three patients were positive and 9 patients were weakly positive for immunoglobulin M antibodies against SARS-CoV-2. Nine patients tested negative for both immunoglobulin M and immunoglobulin G.

### COVID-19 Outcomes

**Figure 2.**
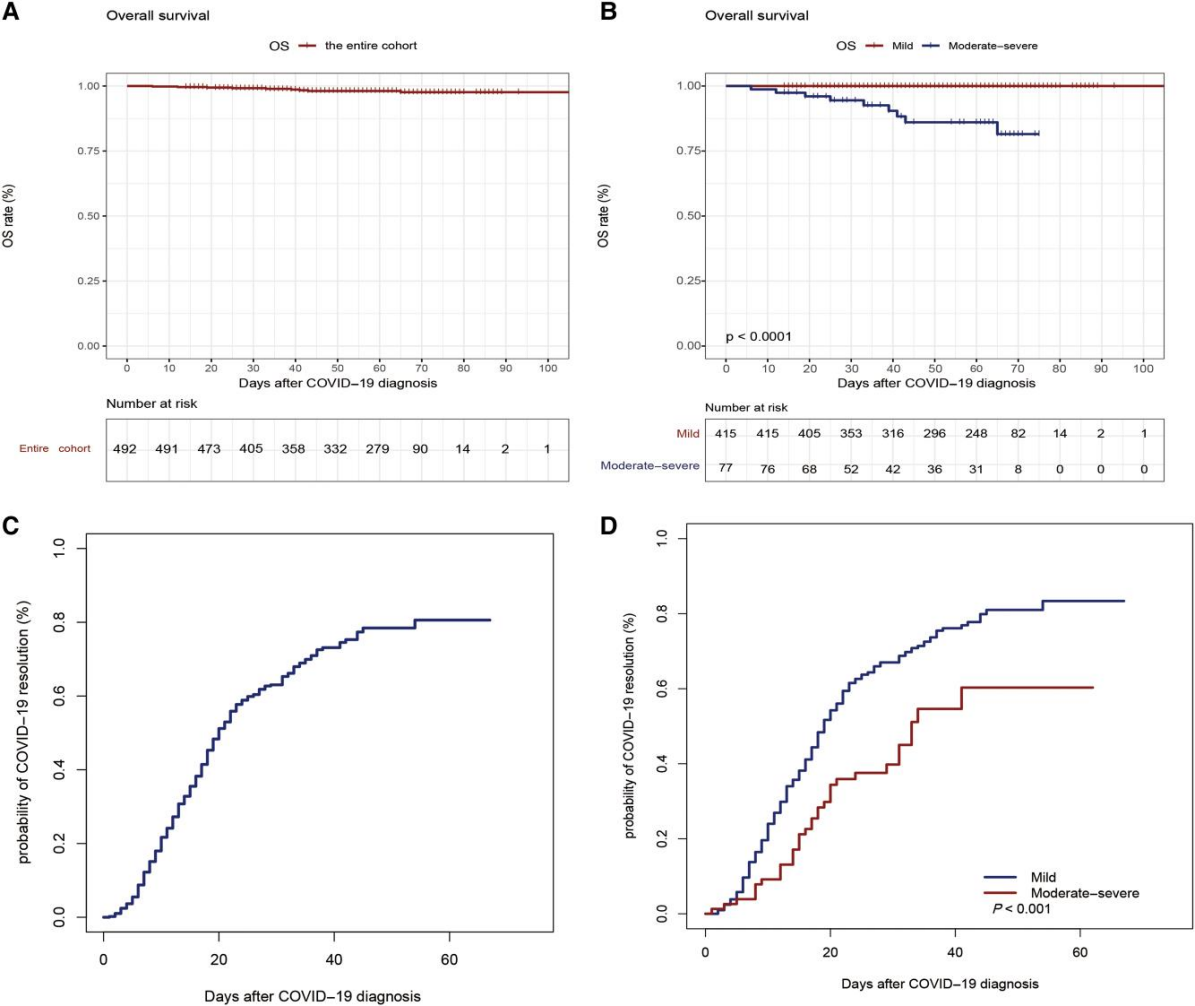
Clinical outcomes of allo-HSCT recipients with COVID-19. OS following COVID-19 diagnosis in the whole cohort (*A*). OS following COVID-19 diagnosis in the mild and moderate-to-severe subgroups (*B*). Probability of COVID-19 resolution in the entire cohort (*C*). Probability of COVID-19 resolution in the mild and moderate-to-severe subgroups (*D*). allo-HSCT, allogeneic hematopoietic stem cell transplantation; COVID-19, coronavirus disease 2019; OS, overall survival.

Among the 492 allo-HSCT recipients, 69 required hospital admission for COVID-19. The COVID-19 duration was 19 (1–67) days for the entire cohort, with 80.1% patients having a disease duration of <30 days. For patients with long-term disease (>30 days), 75, 18, and 5 exhibited mild, moderate, and severe disease, respectively. In the subgroup of mild COVID-19, 11 patients received antiviral treatments. Among 77 patients with moderate-to-severe COVID-19, 35 were administered with antiviral therapies (Table 1). At the last follow-up, 324 patients had clinically resolved COVID-19, 145 were improving, and 23 had ongoing disease. Among the 324 patients with clinical resolution of COVID-19, 220 were tested negative for SARS-CoV-2 on their nasopharyngeal swab samples. The remaining 104 patients did not undergo a nasopharyngeal swab test. The incidence of COVID-19 resolution at 60 days was 80.6% in all the allo-HSCT recipients (Figure 2). The 60-day resolution rates of COVID-19 in the mild and moderate-to-severe groups were 83.4% and 60.3%, respectively (*P* < .001) (Figure 2). Eventually, 9 patients died (median time from COVID-19 diagnosis, 33 days). Among them, 8 had severe COVID-19 and 1 had moderate COVID-19. Six of the 9 patients died of COVID-19 complications, and the remaining 3 patients died because of underlying disease relapse or graft failure. Among the 6 deceased patients with complete remission of underlying disease, 2 received rituximab for Epstein–Barr virus infection at the time of COVID-19 diagnosis. In the entire cohort, the 60-day OS rate was 98.1% (95% CI, 97.0–99.2) (Figure 2). The 60-day OS rates for patients with mild and moderate-to-severe disease were 100% and 86.0% (95% CI, 76.8–95.2; *P* < .001), respectively (Figure 2). The factors associated with survival after COVID-19 diagnosis are summarized in Table 3. Univariate analysis revealed that corticosteroid therapy or immunosuppressive treatment within 3 months before COVID-19 diagnosis and early COVID-19 following allo-HSCT (<6 months) were associated with survival after disease diagnosis.

**Table 3.** Univariate Analysis for Survival After COVID-19 Diagnosis

	Univariate Analysis		
	OR	95% CI	*P* Value
On corticosteroid therapy withing 3 mo before COVID-19 diagnosis (Ref. without)	8.560	1.766–41.494	.008
Interval from allo-HSCT to COVID-19 diagnosis (Ref. ≥6 mo)	3.535	1.760–7.099	<.001
On immunosuppression treatment within 3 mo before COVID-19 diagnosis (Ref. without)	9.837	1.224–79.033	.032
Conditioning regimens (Ref. MAC)	3.562	.890–14.257	.073
BMI			
<18.5	1 (ref)	1 (ref)	
18.5–23.9	0.822	.281–2.407	.721
≥24.0	0.883	.367–2.124	.781
Age at COVID-19 diagnosis (Ref. <50 y)	3..212	.861–11.978	.082
Donor type			
URD	1 (ref)	1 (ref)	
HID	0.738	.168–3.247	.688
MSD	1.320	.463–3.764	.603
History of acute GVHD (Ref. without)	1.325	.331–5.306	.691
History of chronic GVHD (Ref. without)	0.314	.065–1.510	.148
ATG for GVHD prophylaxis (Ref. without)	0.040	.000–195.464	.458

Abbreviations: allo-HSCT, allogeneic hematopoietic stem cell transplantation; ATG, antithymocyte globulin; BMI, body mass index; CI, confidence interval; COVID-19, coronavirus disease 2019; GVHD, graft-versus-host disease; HID, haploidentical donor; MAC, myeloablative conditioning; MSD, matched sibling donor; OR, odds ratio; URD, unrelated donor.

## DISCUSSION

This study presents the clinical characteristics, outcomes, and risk factors for moderate-to-severe COVID-19 in a large cohort of allo-HSCT recipients during the Omicron wave. Overall, 15.6% allo-HSCT recipients contracted moderate-to-severe COVID-19. Remarkably, the 60-day OS rate of patients with COVID-19 was 98.1% (95% CI, 97.0–99.2). The probability of resolution at 60 days post–COVID-19 was 80.6% in allo-HSCT recipients. Patients who received corticosteroid therapy or had COVID-19 within 6 months after HSCT exhibited a higher risk of developing moderate-to-severe disease.

Patients who underwent allo-HSCT, autologous HSCT, and chimeric antigen receptor T-cell therapy demonstrated a higher risk severe COVID-19 and death during the pre-Omicron wave [17, 19, 20]. The clinical characteristics of patients with COVID-19 have been altered because of the advent of Omicron variants involves considerably less involvement of the lower respiratory tract, hospital admissions, and COVID-19 deaths [21, 26]. Here, 19.9% of the patients had long-term COVID-19 (>30 days). Consistent with the outcomes of immunocompromised patients, 25% of patients with COVID-19 continue to exhibit symptoms for >30 days after disease onset [24]. Prolonged COVID-19 is frequent in the general population and in patients with hematological malignancies [27, 28]. Our results revealed that allo-HSCT recipients exhibited a low COVID-19 mortality rate during the Omicron wave, 14.0% patients with COVID-19 required hospitalization, and only 15.6% patients exhibited moderate to severe symptoms. According to a previous study, immunocompromised patients with COVID-19 demonstrated low COVID-19 mortality (1/114) and frequent hospitalizations (20%) [24]. Similarly, a recent study reported that Asian patients who underwent allo-HSCT exhibited a substantially lower mortality rate (7.8%) at 90 days post–COVID-19, and 90.9% patients exhibited mild disease when the Delta and Omicron variants predominated [29]. The favorable outcomes of allo-HSCT recipients may be attributed to the low virulence of the Omicron strain [30].

This study revealed that a shorter interval (<6 months) between allo-HSCT and COVID-19 diagnosis constituted a significant risk factor for moderate-to-severe COVID-19. Similarly, several studies demonstrated that patients who contracted COVID-19 within the first year following allo-HSCT exhibited poorer outcomes during the pre-Omicron wave [17, 20, 31]. In addition, the present study confirmed that corticosteroid use 3 months before COVID-19 onset increased the predisposition to moderate-to-severe COVID-19. A previous study reported steroid use in patients with COVID-19 affected disease severity [17]. Notably, we found that ATG for GVHD prophylaxis increased the likelihood of moderate-to-severe COVID-19 in allo-HSCT recipients. ATG as a GVHD prophylaxis impairs immune reconstitution following allo-HSCT [32] and modulates immune response by interfering with the function of different immune effectors, especially T lymphocytes [33]. Hence, ATG may exert adverse effects on severe COVID-19.

Herein, multivariate analysis revealed no significant association between immunosuppressive therapy and COVID-19 severity. Similarly, according to a 2021 report by the Center for International Blood and Marrow Transplant Research, immunosuppressive agents exhibited no significant influence on severe disease in allo-HSCT recipients with COVID-19 diagnosed before 27 March 2020 [20]. However, a multicenter cohort study in France reported that any immunosuppressive therapy increased the risk of COVID-19 during the first (March–June 2020) and second (August 2020–June 2021) waves [34]. In addition, the German Cooperative Transplant Study Group confirmed that immunosuppression with cyclosporine during COVID-19 diagnosis was a significant risk factor in patients with allo-HSCT were infected with SARS-CoV-2 between February 2020 and July 2021 [31]. Notably, Ljungman et al. reported that the immunodeficiency index increased the risk of mortality rather than ongoing immunosuppression [35]. Presumably, patients in our cohort received different immunosuppressive therapies while maintaining a consistent status of immune cells before COVID-19 diagnosis. This may have contributed to the finding that immunosuppression did not influence COVID-19 severity. Furthermore, this study revealed that COVID-19 exerted no significant influence on immune reconstitution in patients undergoing allo-HSCT, especially in the mild COVID-19 subgroup. Consistent with the results of a previous study, immune profiling revealed reductions and rapid recovery in patients receiving cellular therapy who had COVID-19 [19]. More detailed and comprehensive immune cell monitoring of the peri–COVID-19 period is warranted to determine the effect of immunosuppressive therapy on SARS-CoV-2 infection.

The effect of recipient age at the time of COVID-19 diagnosis on COVID-19 severity remains controversial. Although univariate analysis revealed a significant association between patient age and infection severity, multivariate analysis demonstrated no significant relationship. Nevertheless, according to studies involving allo-HSCT recipients in the pre-Omicron phase, the older age of patients at the time of COVID-19 diagnosis increased the risk of severe COVID-19 [20, 31, 35]. In France, age was a significant risk factor for severe COVID-19 during the second wave and did not exert any effect during the first wave [34].

Although 25.4% allo-HSCT recipients (125) had a history of COVID-19 vaccination before COVID-19 diagnosis, vaccination demonstrated no significant influence on COVID-19 severity in the present study. However, a single-center cohort of 43 allo-HSCT and 34 autologous HSCT recipients revealed that being unvaccinated at the time of disease onset was a risk factor for more severe disease [29]. The effectiveness of COVID-19 vaccination in allo-HSCT recipients is difficult to assess because vaccination data (eg, type and dose of vaccines for each individual) were insufficient in the present study. Prospective multicenter cohort studies are required to identify the risk factors for COVID-19 severity.

This study has some limitations. Considering its retrospective nature, selection (eg, Berkson's bias) and information bias (eg, recall bias) may be present in the present study. First, data regarding SARS-CoV-2 variants, long-term monitoring of immune reconstitution, and COVID-19 vaccine type in each allo-HSCT recipient were limited. Second, COVID-19 treatment information was incomplete as several patients were outpatients, rendering it difficult to estimate the effect of treatment on COVID-19. Third, few patients with severe disease were lost to follow-up, which may have led to the underestimation of COVID-19 mortality.

In conclusion, prolonged COVID-19 was frequent in allo-HSCT recipients. Moreover, they exhibited low rates of moderate-to-severe COVID-19 and mortality during the Omicron wave. The risk of developing moderate-to-severe disease was higher among patients who received corticosteroid therapy, had a history of ATG for GVHD prophylaxis, or contracted COVID-19 early following allo-HSCT compared with others. Preventive strategies are important for allo-HSCT recipients with a high risk of COVID-19. Moreover, prospective multicenter cohort studies are required to determine the risk factors for severe COVID-19 and provide insights into the immune reconstitution.

## Supplementary Material

ofae038_Supplementary_Data

## Supplementary Data

Supplementary materials are available at the *Journal of The Pediatric Infectious Diseases Society* online (http://jpids.oxfordjournals.org). Supplementary materials consist of data provided by the author that are published to benefit the reader. The posted materials are not copyedited. The contents of all supplementary data are the sole responsibility of the authors. Questions or messages regarding errors should be addressed to the author.
