# Supplementary material for: Characteristics of Coronavirus Disease in Allogeneic Hematopoietic Stem Cell Transplantation During the Omicron Wave: A Single-Center Study
Source: Open Forum Infect Dis. 2024 Jan 23;11(3):ofae038. doi: 10.1093/ofid/ofae038 (PMC10932942; doi:10.1093/ofid/ofae038)
Supplement: ofae038_Supplementary_Data [file ofae038_supplementary_data.docx]

**SUPPLEMENTARY MATERIAL**

**Supplementary Table 1** – Baseline clinical characteristics of the 492 allo-HSCT recipients with COVID-19

| Characteristics | N (%) |
| --- | --- |
| Age at COVID-19 diagnosis, median (range), years | 41 (18–71) |
| 18–29 | 101 (20.6) |
| 30–39 | 120 (24.4) |
| 40–49 | 123 (25.0) |
| 50–59 | 109 (22.0) |
| ≥60 | 40 (8.1) |
| Gender, male/female | 245/247 |
| BMI, median (range), kg/m^2^ | 21.7 (13.0–41.4) |
| <18.5 | 84 (17.1) |
| 18.5–23.9 | 285 (57.9) |
| ≥ 24.0 | 123 (25.0) |
| Underlying disease |  |
| AML/MDS | 288 (58.5) |
| ALL | 150 (30.5) |
| Other | 54 (11.0) |
| Donor type |  |
| MSD | 84 (17.0) |
| HID | 344 (70.0) |
| URD | 64 (13.0) |
| Conditioning regimens |  |
| MAC | 426 (86.6) |
| RIC | 66 (13.4) |
| ATG for GVHD prophylaxis |  |
| Yes | 430 (87.4) |
| No | 62 (12.6) |
| CD34+ cells, median (range), ×10^6^/kg | 5.1 (1.18–17.6) |
| MNC, median (range), ×10^8^/kg | 11.96 (2.78–49.4) |
| Neutrophils engraftment, median (range), days | 12 (9-21) |
| Platelets engraftment, median (range), days | 13 (0-42) |
| Year of transplantation |  |
| Before 2019 | 122 (24.8) |
| After 2020 | 370 (75.2) |
| Interval from allo-HSCT to COVID-19 diagnosis, median (range), months | 18.5 (0.8–145.8) |
| < 6 months | 86 (17.5) |
| 6–12 months | 82 (16.7) |
| 12–24 months | 138 (28.0) |
| ≥ 24 months | 186 (37.8) |
| History of COVID-19 vaccination |  |
| Yes | 125 (25.4) |
| No | 367 (74.6) |
| Doses of Vaccination |  |
| 1 | 18 (14.4) |
| 2 | 67 (53.6) |
| 3 | 40 (32.0) |
| Time from the last vaccination to COVID-19 diagnosis, median (range), days | 456 (2-694) |
| On immunosuppression treatment in 3 months before COVID-19 diagnosis |  |
| Yes | 250 (50.8) |
| No | 242 (49.2) |
| On corticosteroid treatment within 3 months before COVID-19 diagnosis |  |
| Yes | 170 (34.6) |
| No | 322 (65.4) |
| GVHD at COVID-19 diagnosis |  |
| Yes | 139 (28.3) |
| No | 242 (71.7) |
| Follow-up time, median (range), days | 62 (6–110) |
| Abbreviations: BMI, body mass index; AML, acute myelocytic leukemia; MDS, myelodysplastic syndrome; ALL, acute lymphoblastic leukemia; MSD, matched sibling donor; HID, haploidentical donor; URD, unrelated donor; MAC, myeloablative conditioning; RIC, reduced intensity conditioning; ATG, anti-thymocyte globulin; GVHD, graft-versus-host disease; allo-HSCT, allogeneic hematopoietic stem cell transplantation; COVID-19, coronavirus disease 2019 | |

**Supplementary Table 2** – Clinical characteristics of COVID-19 patients with monitoring of immune cells.

| Characteristics | Mild COVID-19 (N = 56) | Moderate-severe COVID-19 (N = 20) | *P*-value |
| --- | --- | --- | --- |
| Age at COVID-19 diagnosis, median (range), years | 44 (18–68) | 43 (23–70) | 0.962 |
| Gender, male/female | 27/29 | 10/10 | 0.891 |
| BMI, median (range), kg/m^2^ | 20.7 (14.4–29.2) | 20.8 (14.1–41.4) | 0.925 |
| Underlying disease |  |  | 0.495 |
| AML/MDS | 33 | 11 |  |
| ALL | 13 | 7 |  |
| Other | 10 | 2 |  |
| Donor type |  |  | 0.680 |
| MSD | 5 | 3 |  |
| HID | 39 | 12 |  |
| URD | 12 | 5 |  |
| Conditioning regimens |  |  | 0.547 |
| MAC | 41 | 16 |  |
| RIC | 15 | 4 |  |
| ATG for GVHD prophylaxis |  |  | 0.375 |
| Yes | 51 | 19 |  |
| No | 5 | 1 |  |
| Interval from allo-HSCT to COVID-19 diagnosis, median (range), months | 5.4 (0.8–145.8) | 4.7 (1.2–16.3) | 0.981 |
| Interval from pre-COVID immune cells to COVID-19 diagnosis, median (range), days | 28 (2–171) | 32 (2–160) | 0.675 |
| CD3 (cells/mm^3^) | 742 (6–5951) | 568.5 (7–2759) | 0.409 |
| CD4 (cells/mm^3^) | 116.5 (0–2054) | 91.5 (0–735) | 0.162 |
| CD8 (cells/mm^3^) | 490 (3–3719) | 420 (4–2004) | 0.791 |
| CD19 (cells/mm^3^) | 19 (0–1117) | 14 (0–860) | 0.392 |
| NK (cells/mm^3^) | 151.5 (0–4778) | 86.5 (18–939) | 0.085 |
| Interval from COVID-19 diagnosis to post-COVID immune cells, median (range), days | 40 (2–79) | 36 (9–74) | 0.165 |
| CD3 (cells/mm^3^) | 1018 (80–3419) | 403 (42–2703) | 0.002 |
| CD4 (cells/mm^3^) | 195 (17–1713) | 85 (8–732) | 0.001 |
| CD8 (cells/mm^3^) | 666 (44–2351) | 277 (31–1534) | 0.006 |
| CD19 (cells/mm^3^) | 85.5 (0–550) | 20.5 (0–1016) | 0.010 |
| NK (cells/mm^3^) | 247 (11–996) | 61 (15–420) | <0.001 |
